# Characterizing performance improvement in primary care systems in Mesoamerica: A realist evaluation protocol

**DOI:** 10.12688/gatesopenres.12782.1

**Published:** 2018-01-03

**Authors:** Wolfgang Munar, Syed S. Wahid, Leslie Curry

**Affiliations:** 1 Milken Institute School of Public Health, George Washington University, Washington, DC, 20052, USA; 2 Department of Health Policy and Management , Yale School of Public Health, New Haven, CT, 06520-8034, USA

**Keywords:** El Salvador, Honduras, Primary Care accountability reforms, Primary Care Performance in low- and middle-income countries, Primary Care performance measurement, Realist Evaluation, Results-based financing, Salud Mesoamerica Initiative

## Abstract

**Background**. Improving performance of primary care systems in low- and middle-income countries (LMICs) may be a necessary condition for achievement of universal health coverage in the age of Sustainable Development Goals. The Salud Mesoamerica Initiative (SMI), a large-scale, multi-country program that uses supply-side financial incentives directed at the central-level of governments, and continuous, external evaluation of public, health sector performance to induce improvements in primary care performance in eight LMICs. This study protocol seeks to explain whether and how these interventions generate program effects in El Salvador and Honduras.

**Methods**. This study presents the protocol for a study that uses a realist evaluation approach to develop a preliminary program theory that hypothesizes the interactions between context, interventions and the mechanisms that trigger outcomes. The program theory was completed through a scoping review of relevant empirical, peer-reviewed and grey literature; a sense-making workshop with program stakeholders; and content analysis of key SMI documents. The study will use a multiple case-study design with embedded units with contrasting cases. We define as a case the two primary care systems of Honduras and El Salvador, each with different context characteristics. Data will be collected through in-depth interviews with program actors and stakeholders, documentary review, and non-participatory observation. Data analysis will use inductive and deductive approaches to identify causal patterns organized as ‘context, mechanism, outcome’ configurations. The findings will be triangulated with existing secondary, qualitative and quantitative data sources, and contrasted against relevant theoretical literature. The study will end with a refined program theory. Findings will be published following the guidelines generated by the Realist and Meta-narrative Evidence Syntheses study (RAMESES II). This study will be performed contemporaneously with SMI’s mid-term stage of implementation. Of the methods described, the preliminary program theory has been completed. Data collection, analysis and synthesis remain to be completed.

## Introduction

Improving performance of primary care systems in low- and middle-income countries (LMICs) has been suggested as a necessary condition for the achievement of universal health coverage in the age of the Sustainable Development Goals ^1^. High-performing primary care systems not only are the first point of contact for continuous, coordinated, comprehensive and people-centered health services, but also provide critical preparedness and response to public health threats ^2, 3^.

The Salud Mesoamerica Initiative (SMI) is a multi-country, large-scale primary care performance improvement program. It is the result of a partnership between the governments of the eight Mesoamerican nation-states, the Bill and Melinda Gates Foundation, the Carlos Slim Foundation, the Government of Canada, the Inter-American Development Bank (IADB) and, during earlier stages, the Government of Spain. The program is aimed at improving reproductive, maternal, neonatal and child health outcomes among the poorest, rural populations in participating countries. Intended outcomes include increased availability, utilization, and effective coverage of primary care services and a reduction in preventable health inequities. The program’s approach to performance improvement combines the use of high-powered financial incentives at the government-level and the external verification of public sector, primary care system performance.

Programs and policies aimed at improving the performance of health systems have been at the forefront of many public-sector reforms. Initial waves of reforms in the public sectors of high-income countries were focused on learning and improvement ^4– 7^; subsequent waves of reform targeted public sector accountability and organizational best-practice ^8, 9^. Governments in LMICs adopted and replicated these reforms, oftentimes with the support of multilateral finance institutions and agencies in the official development assistance space.

Several generic types of reforms follow the logic of public-sector interventions ^10^, including (1) political interventions as expressed in policies and regulations; (2) reforms by laws; (3) intervention by audit and inspection, based on continuous evaluation of results and conformity to predefined norms; (4) intervention by management, based on organizational science and management practice, such as continuous quality improvement or change management methods, among others; and, (5) intervention by rationalizing professional behaviors such as the introduction of evidence-based practices and the standard comparison of outcomes by public sector providers.

In LMIC, accountability-driven reforms have flourished under the rubric of Results-based Financing (RBF); the health sector has regularly been at the center of such reforms. The Multilateral Finance Institutions, the Global Fund to Fight HIV/AIDS, tuberculosis and malaria, and the Global Alliance for Immunizations have financed results-oriented, global health programs, some of which have targeted health system improvements.

There are ambiguities in the definitions as well as in the scope and content of RBF programs and policies. In this study protocol, RBF is understood as “any program that rewards the delivery of one or more outputs or outcomes by one or more incentives, financial or otherwise, upon verification that the agreed-upon result has actually been delivered” ^11^; incentives can target health care providers (supply side), households (demand side), or both. Performance-based Financing (PBF), is a prevalent type of RBF in which the incentives are exclusively financial; rewards are only aimed at providers; and the payments are usually adjusted for quality. PBF assumes many forms but, in essence, serves the purpose of reforming the ways in which governments pay health care providers (individuals and facilities) for the provision of services.

Accountability-driven interventions in public sector reforms are designed to reduce the misalignment in incentives between principals (voters, legislative bodies, executive-level leadership, funders, etc.) and their agents (program implementers, providers of care, etc.) ^12, 13^. Such reforms usually assume that incentives and rewards serve as powerful motivators for the achievement of desirable behaviors among utility-maximizing, rational individuals ^14– 16^. These assumptions have conventionally been based on principal-agent theory ^14, 15^, positive agency theory ^16^, and/or rational choice theory ^17, 18^. In recent years, there have been calls for using a more expansive view of human agency when discussing motivation and decision-making. Under such views, humans are not exclusively motivated by rewards and incentives, but can also be driven to action by intrinsic motivators ^19^. This perspective has also influenced contemporary research on PBF in LMIC ^20, 21^.

Most of the primary studies that have assessed RBF and PBF programs in LMIC have characterized the effects of financial incentives on provider-level motivation and behaviors ^21– 31^. However, the evidence on the effects of RBF on large-system reforms targeting government-level improvements is scarce; and studies in LMIC settings are largely absent ^32– 34^.

The most-recent systematic review of performance-based financing programs in LMIC concluded that PBF is not a single type of intervention and that its effects are dependent on the interactions among multiple variables ^31^. Most PBF evaluations to date have used a narrow focus such as characterizing changes in health care outputs, while neglecting most other domains of primary care performance improvement ^35^. Furthermore, the empirical evidence about how PBF leads to changes in attitudes and behaviors among public sector actors is scarce ^35^. Domains that have been under-studied include, among others, whether and how do extrinsic and/or intrinsic motivators affect the behaviors, autonomy and responsiveness of providers and managers of primary care delivery systems; the influence that performance measurement data can have on the behaviors of primary care system actors and stakeholders; and the negative effects of RBF reforms, such as gaming, shirking and cream-skimming.

Public-sector reforms tend to incorporate multiple interventions that generate effects at different levels within organizational hierarchies, and among different actors and stakeholders. Those actors and the environments in which they are embedded interact with each other through time, generating inter-dependencies and, oftentimes, leading to counter-intuitive, emergent, and unintended effects. Furthermore, the implementation strategies and ancillary components of reform programs themselves, such as the provision of technical assistance or change management support, can also trigger system changes that need to be better studied ^31^.

Beyond accountability reforms, studies on performance management and performance assessment have empirically studied improvement-driven public-sector reforms. Studies of such reforms in the public sector of the United States have identified factors that can drive organizational learning and improvement. For instance, Moynihan and Landuyt ^36^ found that the most influential predictors of organizational improvement and learning were the use of work-groups as learning forums; the availability of performance information systems that collect, store and disseminate performance data; the existence of a mission orientation that builds a sense of shared vision for success and common purpose; and the existence of organizational slack, such as time and resources that allow people to think and learn.

Improvement reforms are predicated on the assumption that the continuous collection, availability and analysis of performance data and information would lead to organizational improvement and learning ^7^. However, despite widespread calls for using performance data and information to improve decision-making, the utilization of such data and information can rarely be guaranteed ^37^. Also, little is known about the conditions under which performance measurement work or the mechanisms that lead to system improvement. Studies in evaluation science have addressed these issues ^38, 39^. In this literature, the availability and dissemination of performance evaluation can influence system improvement through multi-level changes on individual, interpersonal and/or collective motivation. Considerable research in evaluation science has been informed by this evidence ^40– 44^. We did not find, however, any study assessing the effects of evaluation results on health system performance improvement in LMICs.

## Study setting

In SMI, governments agree with the IADB to the implementation of up to three consecutive, 18–24 month programs, aimed at achieving a series of progressively complex health targets (including inputs, processes, outputs and outcomes) that are externally verified by the University of Washington’s Institute for Health Metrics and Evaluation (IHME). Participating governments contribute domestic funds *a-priori* to attain the agreed-upon targets; once domestic funds are made available and targets are agreed, SMI matches the domestic contribution with grant financing on a 1:1 ratio. Afterwards, the IADB enters into formal performance contracts with each government. In the contract, SMI commits to reimbursing half of the initially invested domestic funds contingent on the achievement of the agreed-upon performance targets ^45^.

Country-specific performance frameworks with geographical targeting of the poorest, rural populations, were negotiated with each government at the start of the program and have remained stable through time. A pass-or-fail policy was agreed, according to which a government has to achieve 80% or more of the approximately ten (10) targets that make-up any given performance framework to be eligible for the reimbursement of half of the initial domestic contribution. Table 1 lists some of the targets agreed by El Salvador and Honduras.

**Table 1.** Summary of performance frameworks in El Salvador and Honduras.

Indicators	Baseline	Target	Indicators	Baseline	Target
EL SALVADOR			HONDURAS		
*First Phase*			*First Phase*		
Number of families enrolled in Family Health Teams	14,681	38,661	Health centers with permanent availability of micronutrient powder for supplementation at home	0	80%
Number of community health units with supply of four modern family planning methods (injectable, barrier, oral and intra-uterine devices).	11	65	Primary and second care level health units supplied with family planning methods according to ministry of health’s current standard	86.4	90%
Review of national policy for micronutrient products distribution to children aged 6–23 months	No	Yes	Maternal & Child health clinics with permanent availability of medications and inputs necessary for treatment of obstetric and neonatal emergency	62.5	80%
Inclusion in the standard on proper therapeutic dosage of zinc for diarrhea treatment in children under five (20 mg of zinc for 10–14 days with each episode).	No	Yes	Second level health care units with permanent availability of medications, inputs and equipment necessary for treatment of obstetric and neonatal emergency	0	2
Percentage of pregnant women enrolled in the prenatal register who had a prenatal checkup with a physician or nurse before week 12 of pregnancy.	67	77	Maternal deaths reported and investigated according to standards in 2013	N. A.	80%
*Second Phase*			*Second Phase*		
Percentage of women of childbearing age (15–49) currently using (or whose partner uses) a modern contraceptive method.	53.5	60.5	Women (aged 15–49) who received at least four prenatal checkups according to best practices by qualified personnel during their most recent pregnancy in the last 2 years	23.7	33.7
Percentage of women of childbearing age (15–49) who had a prenatal checkup according to best practices with a physician or nurse before week 12 in their most recent pregnancy	47.5	62.5	Women (aged 15–49) whose most recent delivery was attended by qualified personnel in a health unit in the last 2 years	68.6	76.6
Percentage of children aged 6–23 months who had a hemoglobin value of < 110 g/L. (Prevalence of anemia in children aged 6–23 months)	46.5	36.5	Neonates with complications (prematurity, low birth weight, asphyxia and sepsis) managed according to hospital standards in the previous two years	6.9	36.9
Percentage of mothers who gave their children (aged 0–59 months) oral rehydration salts and zinc in the last episode of diarrhea	4.4	24.4	Women with obstetric complication (sepsis, hemorrhage and eclampsia) managed according to national standards in their most recent delivery in the last two years	11	51
Percentage of women of childbearing age (15–49 years) whose most recent delivery was attended by trained personnel in a health unit in the last two years.	86.2	94.2	Mothers who report giving their children aged 6–23 months at least 50 packets of micronutrient powder in the last six months (36m)	0.1	15.1
*Third Phase*			*Third Phase*		
Pregnant women treated at health centers in the last year who had at least one preconception consultation with quality in the year before their pregnancy.	-1	10	Women (aged 15–49 years) who currently use (or whose partner uses) a modern family planning method	66.8	76.8
Percentage of women of childbearing age (15–49 years) currently using (or whose partner uses) a modern contraceptive method.	-1	7 PP	Women (aged 15–49 years) whose most recent delivery was attended by qualified personnel in a health unit in the last two years	68.6	8PP
Women who received postpartum contraceptives in the last year.	-1	15PP	Newborns who received neonatal care within 3 days following birth according to standard in the last two years	-2	8PP
Women with obstetric complication (pre-eclampsia with severe symptoms, hemorrhage and sepsis) treated according to national standard.	-1	25 PP	Women with obstetric complication (sepsis, hemorrhage and eclampsia) managed according to the standard in their most recent pregnancy in the last two years	-2	25PP
Neonates with complications (low birthweight, prematurity, asphyxia and sepsis) treated according to the standard.	-1	25 PP	Neonates with complications (prematurity, low birth weight, asphyxia and sepsis) managed according to hospital level standards in the previous two years	-2	25 PP
Newborns who received neonatal care after birth according to the standard in the last two years.	-1	80%	Prevalence of anemia in children aged 6–23 months (Children aged 6–23 months with hemoglobin levels < 110 g/L)	35.3	25.3

As the agency in charge of external verification of government performance, IHME conducts a full-scale quantitative measurement that follows SMI’s sequential process of implementation. Before each country program starts, a baseline is collected and its results disseminated. After that, at the end of each 18 to 24-month implementation projects, IHME collects household and facility-based data to evaluate the achievement of agreed-upon results. Phase 1 programs started in a staggered fashion in 2011; phase-2 programs will finish during 2017; a third and final phase will start in 2018 and go into 2020. Program targets during phase 1 were focused on adherence to protocols, availability of resources and, in general, structure and process performance. During phase 2, targets were focused on outputs, and phase 3 will be centered on health outcomes, including but not limited to, coverage of exclusive breastfeeding, increased modern contraceptive prevalence rates, effective coverage of antenatal care and institutional deliveries, post-partum and post-natal care coverage, and in some cases, reductions in the prevalence of anemia and effective coverage of measles vaccination, measured in blood ^46^. After each round of performance evaluation, results are aggregated and disseminated in each country through policy dialogue workshops convened by the government and involving the IADB and IHME.

**Figure 1.**
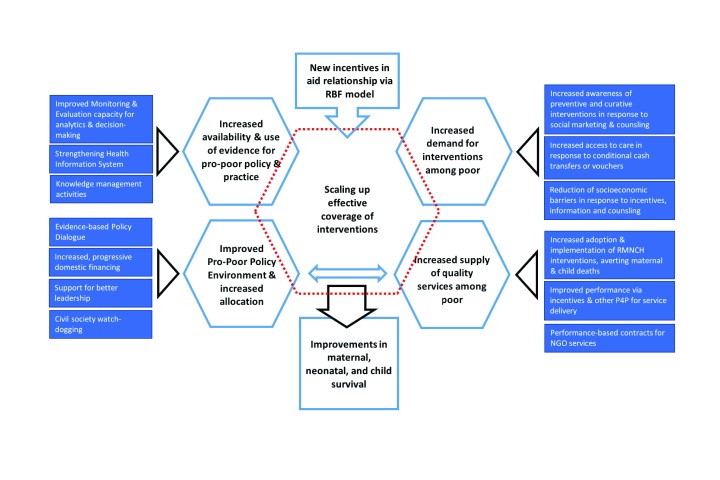
SMI initial theory of change.

SMI’s original theory of change ( Figure 1) hypothesized that the use of supply-side financial incentives directed to central-level ministries in each participating government (Ministries of Finance and Health) would focus their attention on accounting for the achievement of their own agreed-upon health targets. The success of this hypothesis rested on four causal pathways. The first established that the three consecutive, biannual rounds of external verification of performance by IHME would generate sustained pressure on governments for the production of health results. The second pathway proposed that ongoing dialogic, participatory dissemination of data, information and evidence would lead to progressive improvements in the quality of care services and improved, aggregate performance in each participating country’s primary care system. Anticipated population-level health effects were also contingent on increasing domestic pro-poor health spending and expanding the demand for high impact health interventions among beneficiary populations.

While the program’s original theory of change identified several causal pathways, it did not explain *how* its main interventions would trigger outcomes, nor did it provide, either, *a-priori* explanations about the role that each country’s policy context could play in moderating the effects of program interventions. The SMI partnership appears to have embraced a high degree of flexibility in implementation to facilitate governmental buy-in. In 2011, the partners agreed on a set of common principles including a focus on external and independent measurement of results, accountability and transparency, and country ownership. These principles established the institutional boundaries that, in turn, allowed the IADB to negotiate country-specific performance contracts, results frameworks and evaluation plans with each participating government. They also granted implementing partners a high degree of flexibility in the design of each country’s multi-phased implementation plans and, also, led to performance contracts based on a few high-order principles (country ownership, a focus on results, pro-equity, cost-effective interventions, measurability and transparency) that were originally agreed among the funders and the IADB, and reflected in the program’s operating model.

In the two countries under study, El Salvador and Honduras, the program’s focus on country ownership led to each government deciding how to best deploy SMI’s non-reimbursable resources and their own domestic financing to increase the likelihood of achieving programmatic success. For instance, El Salvador had undergone a health system reform in the early 2010s, which coincided with the beginning of SMI implementation. The government decided to focus its targets on results that leveraged one of the reform’s central tenets, the provision of universal primary care services through community-centered, Family Health Teams ^47^. Honduras, in turn, had started in the late 2000s a large-scale pilot of contracting-out and pay-for-performance in the delivery of primary care services ^48^. The government thus decided to leverage its own performance-driven policies and programs and has thus implemented SMI in municipalities that had already acquired experience with RBF.

## Methods

### Methodological approach

The evidence gaps identified in our literature review have led to recent calls for new approaches in the evaluation of complex public-sector reforms, such as PBF ^30, 35, 49, 50^. It has been argued that realist evaluation provides a valuable and relevant approach for assessing interventions that involve changing human decisions and actions ^51, 52^. Realist evaluation is a form of theory-driven inquiry based on the premise an evaluation needs to answer “ *what worked, how, in what circumstances and for whom*”, rather than the conventional question “ *Did the program work?*” ^52^. The appeal of this approach, compared to other theory-driven methods, lies in its explicit foundations in critical realism – an epistemology located between positivism and relativism ^52^. Such perspective contends that program interventions bring about social change through underlying, usually hidden causal mechanisms, and considers the role of context as indispensable in explaining causality.

This study addresses two research questions: (1) What are the effects of using supply-side financial incentives on the performance of the primary care systems in Honduras and El Salvador? How are those effects produced? Under what contextual factors are these effects produced in each country? And, (2) What are the effects of continuous external verification of performance in the two countries under study? How are those effects produced? Under what contextual factors are these effects produced in each country?

While there is no single way to implement a realist evaluation study, as the experience with its use and applications grow, various authors have adopted and adapted Pawson and Tilley’s approach and identified a series of steps that are described below ^52– 57^.

***Developing a preliminary program theory.*** Realist evaluation starts with the development of a program theory that serves as a hypothesis about the ways in which outcomes are produced through the interaction between interventions and context conditions, and mediated by hidden, not-observable mechanisms. The latter have been defined as the ideas and opportunities triggered among program actors and stakeholders in response to program interventions ^57^. The process of testing and refining program theories usually relies upon quantitative and qualitative methods and culminates with a refined program theory ^53^.

This stage in the study was completed through complementary approaches, including (1) review of program design documents; (2) discussions with program designers to gain in-depth understanding of the original causal links between program interventions and expected outcomes; (3) scoping review of the literature, focused on identifying theories and empirical evidence addressing similar processes of primary care system change; and, (4) facilitation of a workshop with IADB stakeholders, which helped understand their assumptions about how program interventions effects could be produced in the two countries under study. This process of making explicit the assumptions held by program stakeholders before data is collected is an essential aspect in realist evaluation. As a result of these various activities, the research team formulated a preliminary program theory.

In a separate, ongoing study we will perform a realist synthesis of performance improvement; performance measurement/evaluation; and, results- and performance-based financing. The process started with a scoping review of social science theories related to the two research questions and led to the mapping and synthesis of theories explaining the contextual factors and causal mechanisms of relevance to this study protocol. This will be followed by a search for primary studies, systematic reviews, realist evaluations and realist syntheses on the themes above and as required by our research questions. The search for the scoping review was done on Science Direct, JSTOR, and Goodle Scholar using a snowballing technique. The theories that were mapped are summarized in Supplementary File 1.

**Figure 2.**
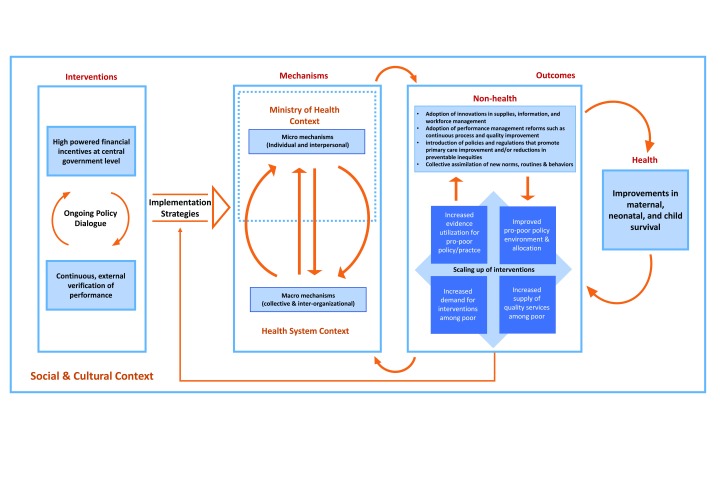
Preliminary program theory.

Based on a synthesis of the results from the scoping review, and informed by the knowledge acquired from the stakeholder workshop and the document review, a preliminary program theory was developed ( Figure 2). It is summarized below as a series of inter-linked propositions:

- The use of (1) high-powered, supply-side financial incentives aimed at central-level government actors and stakeholders (intervention 1) and the implementation of continuous, external evaluation and verification of primary care service performance (intervention 2) supports country priorities through continuous policy dialogue, technical support, and purposive dissemination of performance results (implementation strategy);
- Leading to the adoption of innovations in supplies, information, and workforce management (outcome 1); the adoption of performance management reforms such as continuous process and quality improvement (outcome 2); the introduction of policies and regulations that promote primary care improvement and/or reductions in preventable inequities (outcome 3); and, improved, population-level health outputs and outcomes (outcome 4).
- The behavioral changes listed above occur at various levels within the primary care system, as follows;
  - At the individual level, they satisfy psychological needs such as autonomy, competence and relatedness and/or the need to upgrade or improve personal goals and self-efficacy (individual-level mechanisms) ^58– 63^;
  - At the interpersonal level, because of the aggregate internalization by multiple individual actors and stakeholders, of changes in ideas and opportunities; and/or through a growing sense of public service and/or community service (individual and interpersonal mechanisms) ^19, 39^.
  - Collective level changes could also be triggered whereby the ideas and opportunities of a sufficiently large number of individual actors internalize or assimilate new norms, routines and behaviors which, in turn, spread across inter-organizational and social networks ^64, 65^, leading to the emergence of new organizational culture and collective behavior (outcome);
- Collective inter-organizational-level changes may further lead to the institutionalization and collective assimilation of aggregate individual- and interpersonal-level behaviors through imitation and/or the adoption of new professional and cultural norms, and/or innovative, pro-performance policies (outcome) ^33^ thus, increasing the likelihood of the production of population-level health effects (outcome) and, potentially, transforming the primary care system in a sustained fashion (outcome) ^32, 33^.

Global, institutional, and organizational contextual conditions are also needed for the attainment of program outcomes and for the triggering of the above mechanisms. They include, at the global and sub-regional levels, the existence of favorable conditions such as influential issue-specific global agendas that match existing governmental priorities or a history of interactions between national health agencies and their agendas, and between those and official development aid agencies and their agendas ^66– 69^. At the country-level, the availability of solid institutional environments (laws, regulations, ongoing public-sector reforms, etc.) can create windows of opportunity for the introduction of policy innovations and, also, facilitate convergence between domestic policies and programs, and the externaly-funded interventions. Finally, pre-existing environmental conditions, such as the organizational capacity to absorve new knowledge or the presence of climates that support and enable change, have also been associated with increased assimilation of service innovations ^70, 71^ and need to be considered in the characterization of context.

***Study design.*** In this step the preliminary program theory will be tested, further developed, and validated or rejected. A multiple case-study design with embedded units with contrasting cases was selected ^72^. The contrasting case approach aligns well with the proposition that the two different country contexts in Honduras and El Salvador can trigger to-be-identified mechanisms that generate program results. Given the system-wide and reinforcing effects of the two interventions under study, we define each country’s primary care system as the unit of analysis. Within each country, at least two high-performing municipal-level primary care delivery systems will be analyzed as embedded sub-cases, each with its unique contextual and service delivery structure.

This evaluation is an 18-month study running from May 2017 to December 2018, and executed contemporaneously with SMI’s mid-term stage of implementation. The study seeks to maximize diversity in institutional and policy context to increase the likelihood of identifying variations in policy and program conditions and characterizing the process of change generated to date by the program in one low- and one middle-income country, respectively Honduras and El Salvador. Both countries have to date been exemplars of high-performance, which in SMI is defined as the continuous achievement of 80% or more of the targets agreed between each government and the IADB, and externally verified by IHME.

In each country, the study will assess the context, interventions, implementation approaches, and program effects, intended and otherwise. At the central level of government, the study will characterize program antecedents, policy and organizational context, primary care system’s stewardship and policy-setting, and overall program management and implementation. At the local, municipal level, it will explore primary care delivery through Family Health Teams in El Salvador, and on public as well as non-profit, pay-for-performance providers. Primary data collection will include the methods described below.

***Data collection methods.*** Realist evaluation is method neutral, and the nature of the research, the evaluation questions and the preliminary program theory determine the choice of study design and methodology ^52, 57^. The primary data collection methods to be used in this study include in-depth interviews, non-participatory obervation, and document review.

In-depth interviews with key informants in each country will be conducted to identify individual, inter-personal and collective or organizational factors that may affect primary care system performance in each country under study. These interviews will also be used to elicit contextual elements that could act as barriers or facilitators for the delivery of SMI’s interventions. In this study, we aim to gain a high-level understanding of the causal mechanisms and pathways of performance improvement, as reflected in the preliminary program theory. SMI intervenes at the central as well as local levels of the primary care system, generating hypothesized feedback effects between both. The evaluation aims require the characterization of the interactions and inter-dependencies that occur among multiple actors in the primary care system; this would allow resulting data to help explain the complex nature of the process of performance improvement, and ultimately, help the team validate or revise the preliminary program theory. Accordingly, in-depth key informant interviews will be conducted with four sets of actors: (1) Country policy- and program implementation actors in Honduras and El Salvador; (2) Health care providers at primary care facilities in Honduras and El Salvador; (3) Performance verification and evaluation stakeholders at IHME; and, (4) Program designers at the IADB. Key informants will be recruited using a purposeful sampling approach ^73^. Subsequently, the sample will be snowballed from the initial set of informants.

The study’s sample size cannot be determined *a-priori*, but we expect to conduct approximately 80 key informant interviews, which will ultimately be determined based on theoretical saturation ^74^. Respondents will be invited to participate voluntarily in the study; no compensation will be provided for participation. Interview guides will be used to conduct in-depth interviews; a series of probes will also be developed *a-priori* ( Supplementary File 2). Interviews with country actors and stakeholders will be conducted in Spanish by bilingual members of the research team; IADB and IHME respondents will be interviewed in English. All interviews will be recorded and transcribed verbatim and, when applicable, professionally translated into English.

To document the process of policy dialogue, the study will use non-participant observation during the dissemination of the external verification of performance for the second phase of the program, in early 2018. The research team will document the process followed in the policy dialogue session, the agenda, components and intended objectives, the sequence of events that transpire following the results, and the reactions and actions by country actors and stakeholders. Summary memos of the observations will be generated to be maintained in the project files.

To further understand policy and program context, the study will review key program documents pertinent to the design, implementation and evaluation of SMI interventions in El Salvador and Honduras. Specific attention will be given to documenting the policy and program context in each country, identifying the implementation strategies in each country, assessing performance and evaluation frameworks, and identifying secondary data sources that could be used for further triangulation during the data analysis stage. A complete list of reviewed documents will be maintained, and included as a supplemental file with the final report of findings.

### Data analysis

Data analysis of the in-depth key informant interviews will be conducted using an integrative methodology that merges both inductive and deductive approaches ^74^. We will construct a set of *a-priori* codes drawing from the realist evaluation context, intervention, mechanism, and outcome structure, relevant theoretical literature domains, the stakeholder workshop, and the document review described above. This will be combined with emergent inductive codes identified from a rigorous open coding process.

In an initial stage of data analysis, two coders will analyze a sub-set of transcripts in an iterative and systematic manner using the constant comparison method, and afterwards finalize the codebook through negotiation ^75^. Subsequent transcripts will be coded by three experienced coders using the final codebook.

The coded data will be appraised using two complementary analytic approaches. The research team will use iterative conceptual and pattern coding to identify major emergent inductive themes. At the conclusion of the process, the codes will be arranged into the four major categories of context, intervention, mechanism, and outcomes. The team will scan *within* each category, “vertically”, to identify commonalities and thematic elements, e.g. multiple combinations of contexts that could facilitate/inhibit the interventions; or a confluence of interventions that are catalytic and reinforce one another, etc. Furthermore, the data will be analyzed *across* categories, or “horizontally,” to identify causal patterns whereby certain outcomes are interrelated to program interventions that trigger mechanisms among primary care system actors under specific contextual conditions. We expect these two analytic approaches to be complementary, and to allow building context-mechanism- outcome (CMO) configurations that will then be gauged to determine which patterns plausibly explain how each intervention generated the observed effects, expected and otherwise. The final thematic structure will be used to refine the preliminary program theory. Data analysis will be done with nVivo Version 11 for Mac.

Evaluation results will be completed by integrating findings from the different data collection methods (interviews, notes from non-participatory observations, and secondary document analysis) to confirm, reject or further develop the preliminary program thyeory and the causal patterns identified. The findings will also be contrasted with secondary quantitative and qualitative data sets collected by IHME and others and with social science literature in search for mechanism-oriented theory that may provide explanation for the emerging causal patterns. The results will be a series of CMO configurations that are backed up by the empirical data that provide plausible causal explanations for the observed findings.

***Synthesis and refined program theory.*** In this step the research team will link the emergent CMO conﬁgurations to the preliminary program theory, leading to the adoption, modification, or rejection of the preliminary program theory and will, then, formulate plausible explanations of how and why high-powered, supply side incentives and external verification of performance generate the observed results. The resulting explanations for the observed program effects will then be compiled in the form of narrative summaries, tables, and/or causal loop diagrams. The end of the study product is a final, refined program theory.

Study findings will be published in peer-reviewed periodicals and disseminated locally among policy-makers in the two countries to be studied. The presentation of findings will be made following the Realist And Meta-narrative Evidence Syntheses study (RAMESES II) that was designed to provide guidance on quality assurance and uniform reporting and improve quality and consistency in the reporting of realist evaluations ^76, 77^.

### Quality control

A set of measures will be taken to increase the validity of the study in terms of reflexivity, credibility and confirmability, and enhance the trustworthiness, transparency, and accountability of the research ^78^. All researchers will engage in the introspective practice of maintaining ‘personal biases memos’ to make explicit all self-identified biases and pre-conceptions that may effect the research process ^78^. All analytic decision notes and memos, biases memos, document analysis syntheses, interview guides, research team meeting agendas and minutes, and analysis outputs including coded transcripts, conceptual frameworks, tables, etc. will be preserved to provide a verifiable audit trail.

## Discussion

The refined program theory and CMO configurations resulting from this study have several anticipated uses and applications. Program implementers like the IADB and Salvadorian and Honduran government actors, for instance, can use the findings to consider introducing adjustments in SMI’s implementation during its third and final phase (2018–2020). Also, given the study’s focus on exploring the linkages between ongoing, pre-existing policy mandates and priorities, it is plausible to expect study findings to be of relevance to further improve the evaluation and subsequent re-design of domestic health policies in El Salvador and Honduras. Program evaluators like IHME and other research groups, can use program findings to enhance ongoing evaluation activities or to inform the design of new evaluations that deepen one or more of the various casual patterns identified. For example, our research team intends to use the emerging CMO configurations in the area of performance management to inform the design of a new study to explore whether and how SMI quality improvement interventions produce gains in primary care performance. We also expect program findings to set the stage for further realist evaluations in other large-scale primary care reform in contexts other than Mesoamerica.

Another source of complexity in this study arises from the significant evidence gaps that we identified and from the multiple fields that would need to be rigorously studied to properly address the various outcomes generated by accountability and performance management reforms. As discussed before, such outcomes can occur at different levels of analysis (individual, organizational and collective) and in different contexts (high- as well as LMIC). Not only does this type of research demand inter- and multi-disciplinary capabilities within research teams, but it also calls for rigorous, systematic assessment and mapping of the evidence gaps ^79, 80^. Theory-based program evaluations of primary care performance improvement would also benefit from the publication of realist syntheses that rigorously appraise the literature in search of context-mechanism-outcomes and program theory ^81, 82^. Such studies would not only facilitate the work of research teams currently addressing primary care performance improvement research, but would also strategically shape future health system research agendas, particularly in LMIC.

The research team has faced several challenges in shaping this study’s hypotheses, or preliminary program theory. Many of these challenges are common to other realist evaluations and have been discussed elsewhere ^53, 83^. One such challenge pertains to settling on an unambiguous and precise definition for what constitutes a mechanism. Several definitions in the literature are of a descriptive nature and focus on well-known features of mechanisms such as them being unobservable, context-specific and being able to generate effects. We settled on a definition of mechanisms as the ideas and opportunities triggered among program actors and stakeholders in response to program interventions ^57^. Such an approach is consistent with a view of social change according to which the beliefs, choices and opportunities of individual actors and the interactions among them (micro-level) are the main drivers of social change. This approach also recognizes that the “macro,” social and cultural environment in which these individual actors are embedded can shape social change by means of the internalization of collective values, norms and institutions among individual actors ^84, 85^.

Based on these considerations, this study aims to, first, explore plausible causal explanations based on individual or group-based ideas and opportunities among program actors and stakeholders and, second, to ground those observations on an understanding of the policy and program context in which those actors and stakeholders are embedded. Therefore, we expect that any explanation of primary care system performance improvement needs to address both individual, micro-level, as well as collective, macro-level properties that “are not meaningfully attributed to individuals” ^84^. Three specific types of mechanisms that explain social change are thus of interest to this study ^86^.

*Situational mechanisms* refer to the macro, organizational-level environment in which SMI actors and their social interactions or linkages occur, including domestic policy-makers, ministry of health managers and primary care providers, among others. It also includes SMI stakeholders such as the implementation agency (the IADB) and the external evaluators of performance (IHME). This type of mechanism operates in the direction from macro environment to individual actors (macro-to-micro change). *Action-formation mechanisms* are those that explain how actors’ ideas and opportunities influence individual behaviors across the primary care system. In this type of mechanism, the interaction between program interventions and context, trigger changes in individuals’ ideas and opportunities that further influence others in the same social system. This type of mechanism can generate effects that spread from an individual actor to additional actors (micro to micro change). Finally, *transformational mechanisms* provide explanations of how the sum of new behaviors of multiple individual actors in the primary care system bring about change across the entities that conform the primary care system’s macro environment such as norms and institutions (micro-to-macro social change).

Due to limitations in scope, this study can only plausibly characterize some of the “macro” and “micro” mechanisms triggered during SMI’s initial stages up until its current, mid-term stage (2011–2017). It is also plausible to characterize intended and unintended effects generated during this same period. Findings can also be used identify propositions about downstream effects that could occur or not during the final implementation phase (2018–2020) and about the mechanisms that could help sustain desirable effects after implementation ends. Longer-term, transformational mechanisms, their anticipated effects and the underlying context-mechanism-outcome configurations will, however, remain outside our scope of work.

Another challenge in this study refers to the contested nature of the current definitions used to characterize the interventions that conform RBF, PBF or any of the various reforms that use supply-side incentives to drive accountability in public sector actors and, as is the case in SMI, across the entire primary care system. Like others before us, we settled on the definitions provided by Musgrove ^11^, but we remain cognizant of the fact that PBF is not a single intervention and that its “ancillary” components can themselves generate system effects ^31^. In this respect, this study frames SMI interventions as generic types of public-sector reforms aimed at inducing accountability and organizational improvement and learning. Given that the challenges in defining what these large-scale reforms contain in specific contexts –both in high and well as less-developed nations- the use of a realist evaluation approach will likely contribute to the theorizing of how and why specific contexts generate health and non-health effects in primary care performance management reforms.

Finally, given the method-neutrality that is central to realist evaluations, this study also faced the challenge of settling on a final sequence and content of research methods and activities. We decided to, first, follow the steps described by Vareilles and Marchal in relatively similar realist evaluations and studies ^53, 57^, but also relied on the Realist and Meta-narrative Evidence Syntheses study that provides guidance on how to improve quality and consistency in the reporting of realist evaluations ^77^. By aligning protocol design to these guidelines, we expect that the furture publication of this study’s findings will adhere to current best practice.

## Ethical statement

The study’s protocol was reviewed and declared exempt by the George Washington University’s Institutional Review Board (study number 041733). The Ministries of Health of El Salvador and Honduras were informed of the proposed research by the IADB and provided written approval for the research activities.

Ethical approval documentation will be made available on request. The study will employ scrupulous adherence to the highest ethical standards, and current international and local legislation pertaining to research governance. The data collection will operate under explicit informed consent, which will be preserved in study records. Respondents will be given the choice to provide consent verbally on tape before the interviews, or in writing. To maintain anonymity, respondents will reserve the right to review the study outputs and withdraw consent if necessary. All identifying information will be removed from transcripts and stored separately with access restricted to the research team. All transcripts will be stored electronically in password protected cloud services, and physical documents will be securely stored at George Washington University, Milken Institute School of Public Health.

## Supplementary material

Supplementary File 1: Social science theories mapped in the scoping literature review.

Supplementary File 2: In-depth interview guides.
