# Supplementary material for: Characterizing performance improvement in primary care systems in Mesoamerica: A realist evaluation protocol
Source: Gates Open Res. 2018 Jan 3;2:1. [Version 1] doi: 10.12688/gatesopenres.12782.1 (PMC5801599; doi:10.12688/gatesopenres.12782.1)
Supplement: Supplementary file 1 [file gatesopenres-2-13842-s0000.tgz › d0082959-cba4-4853-914d-064e288610d2.docx]

**Supplementary File 1 -** **Theories that undergird the study’s preliminary program theory**

The starting point for our scoping review was a realist synthesis on large-system transformations in health care interventions in high-income countries (Best, Greenhalgh *et al*. 2012). Such system transformations are aimed at coordinated, system-wide change affecting multiple organizations and care providers, with the goal of significantly improving the efficiency and quality of health care delivery and population-level outcomes. Evaluations of such complex programs are scarce and have mainly been conducted in industrialized settings (Greenhalgh, Humphrey *et al*. 2009). Large-scale system transformations provide, however, a close match in goals and approaches to system change to SMI’s focus on improvement, accountability, and effective coverage at the population level.

In our scoping review, we used a snowballing technique and conducted iterative searches using Science Direct, JSTOR and Google Scholar. The aim of such review was to map existing social science theory and evidence on the two research questions through the identification, review and synthesis of key concepts, mechanisms and contextual factors identified in previous evaluations with a particular, but not exclusive, focus on realist evaluations, systematic reviews, and realist syntheses of large-scale health care systems transformations in any context.

The scoping review elicited several mechanism-informed theories of public sector performance-based incentives and measurement, including (1) the rational actor theory; (2) the principal-agent theory; (3) stakeholder theory (e.g.: accountability theories); and (4) cybernetics-systems theory (Schwartz 2010). In recent years, primary studies, systematic reviews, realist evaluations and realist reviews have also addressed the performance and motivation of front-line workers and performance-based financing in LMIC settings. The latter provided deeper understanding about the causal mechanisms linking context, cognition, motivation and human action and, also, helped us identify many of the theories of relevance to explain causal mechanisms of relevance this study protocol, particularly, (1) self-determination theory; (2) goal-setting theory; and (3) self-efficacy theory.

Through the scoping review, two additional strands in the literature were found to be of relevance: (1) in evaluation science, studies that address the influence of evaluation results; and (2) in the field of diffusion of innovation, studies that address the adoption and use of new knowledge, data, information and evidence. These two strands in the literature have identified mechanism-oriented frameworks that have been empirically tested in high-income countries and, to a much lesser extent, in LMICs. The theories underpinning these types of studies include (1) neo-institutional theory; (2) implementation studies in political science and public management reforms; and, as an over-arching theory, (3) the theory of diffusion of innovations. The latter is also relevant to the diffusion of policy innovations and the “transmission” of global-to-local issue agendas in global health.

Table 1 summarizes the theoretical underpinnings from which we elicited a preliminary program theory. The extent to which this literature will be applicable to developing-country settings will be tested through our research and it will be further complemented by through the present study protocol and by a complementary realist synthesis.

Table 1 - Theories elicited in the scoping review

| Mechanism-driven Social Science theories | Key References (With reference to causal mechanisms) |
| --- | --- |
| Rational Actor Theory | (Monroe and Maher 1995, Streib and Poister 1999, Wang 2002) |
| Principal-Agent Theory | (Grossman and Hart 1983, Perry, Engbers *et al*. 2009) |
| Stakeholder theory | (Boyne 2002, Boyne, Gould-Williams *et al*. 2005, Meier, O'Toole *et al*. 2007, Andrews and A. 2010) |
| Cybernetics-system theory | (Kravchuk and Schack 1996) |
| Evaluation use theory | (Mark and Henry 2004) |
| Neo-institutional theory | (Scott 2000, Macfarlane, Exworthy *et al*. 2011, MacFarlane, Barton-Sweeney *et al*. 2013) |
| Diffusion of innovation theory | (Rogers 2003, Greenhalgh, Robert *et al*. 2004, Greenhalgh, Humphrey *et al*. 2009, Best, Greenhalgh *et al*. 2012) |
| Policy diffusion (diffusion of innovations and political science) | (Weyland 2005, Weyland 2005, Shiffman 2007, Shiffman 2009) |
| Research Utilization theory (diffusion of innovations) | (Huberman 1990, Huberman 1994, Bero, Grilli *et al*. 1998, Rogers 2003, Greenhalgh, Robert *et al*. 2004, Howlett 2009, Lavis, Oxman *et al*. 2009, Lavis, Wilson *et al*. 2009, Ottoson 2009, Chunke, Huang *et al*. 2010, Boyko 2012) |
| Knowledge Translation literature (linguistics) | (Armstrong, Waters *et al*. 2006, Tetroe 2007, Boyko 2012) |
| Knowledge utilization theory | (Johnson 1998, Weiss 1998, Landry, Amara *et al*. 2001) |
| Implementation theory (political science and public administration) | (Johnson 1998, O'Toole Jr 2000, Landry, Amara *et al*. 2001, DeLeon and DeLeon 2002, Henry and Mark 2003) |
| Theories of human action such as self-determination theory; goal-setting theory; and self-efficacy | (Bandura 1977, Deci and Ryan 1985, Ryan and Deci 2000, Locke and Latham 2002, Gagné and Deci 2005, Latham, Borgogni *et al*. 2008) |

**References**

Andrews, R. and B. G. A. (2010). "Capacity, Leadership, and Organizational Performance: Testing the Black Box Model of Public Management." Public Administration Review **70**(3): 443-454.

Armstrong, R., E. Waters, H. Roberts, S. Oliver and J. Popay (2006). "The role and theoretical evolution of knowledge translation and exchange in public health." J Public Health (Oxf) **28**(4): 384-389.

Bandura, A. (1977). "Self-efficacy: towards a unifying theory of behavioural change." Psychol Rev **84**: 191 - 215.

Bero, L. A., R. Grilli, J. M. Grimshaw, E. Harvey, A. D. Oxman and M. A. Thomson (1998). "Closing the gap between research and practice: an overview of systematic reviews of interventions to promote the implementation of research findings." British Medical Journal **317**: 465-468.

Best, A., T. Greenhalgh, S. Lewis, J. S. E., S. Caroll and J. Bitz (2012). "Large-System Transformation in Health Care: A Realist Review." The Milbank Quarterly **90**(3): 421-456.

Boyko, J. A., *et al*. (2012). "Deliberative dialogues as a mechanism for knowledge translation and exchange in health systems decision-making." Social Science & Medicine **75**(11): 1938-1945.

Boyne, G., Gould–Williams, J., Law, J., & Walker, R. (2002). "Plans, performance information and accountability: the case of best value." Public Administration **80**(4): 691-710.

Boyne, G. A., J. S. Gould-Williams, J. Law and R. M. Walker (2005). "Explaining the adoption of innovation: An empirical analysis of public management reform." Environment and Planning C: Government and Policy **23**(3): 419-435.

Chunke, S., J. L. Huang and N. Contractor (2010). "Understanding structures, antecedents and outcomes of organisational learning and knowledge transfer: a multi-theoretical and multilevel network analysis." European Journal of International Management **4**(6): 576-601.

Deci, E. L. and R. M. Ryan (1985). Intrinsic motivation and self-determination in human behavior. New York, Plenum.

DeLeon, P. and L. DeLeon (2002). "What ever happened to policy implementation? An alternative approach." Journal of Public Administration Research and Theory: J-PART, **12**(4): 467-492.

Gagné, M. and E. L. Deci (2005). "Self‐determination theory and work motivation." Journal of Organizational Behavior **26**(4): 331-362.

Greenhalgh, T., C. Humphrey, J. Hughes, F. Macfarlane, C. Butler and R. Pawson (2009). "How Do You modernize a health service? A realist evaluation of whole‐scale transformation in London." Milbank Quarterly **87**(2): 391-416.

Greenhalgh, T., G. Robert, P. Bate, O. Kyriakidou, F. Macfarlane and R. Peacock (2004). How to spread good ideas. A systematic review of the literature on diffusion, dissemination and sustainability of innovations in health service delivery and organisation. Report for the National Co-ordinating Centre for NHS Service Delivery and Organisation R & D (NCCSDO). London, University College.

Grossman, S. J. and O. D. Hart (1983). "An analysis of the principal-agent problem." Econometrica: Journal of the Econometric Society **51**(1): 7-45.

Henry, G. T. and M. M. Mark (2003). "Beyond use: Understanding evaluation’s influence on attitudes and actions." American Journal of Evaluation **24**(3): 293-314.

Howlett, M. (2009). "Policy analytical capacity and evidence-based policy-making: Lessons from Canada." Canadian Public Administration **52**(2): 153-175.

Huberman, M. (1990). "Linkage between researchers and practitioners: a qualitative study." American Educational Research Journal **27**(2): 363-391.

Huberman, M. (1994). "Research utilization: the state of the art." Knowledge Policy **7**(4): 13 - 33.

Johnson, R. B. (1998). "Toward a theoretical model of evaluation utilization." Evaluation and Program Planning **21**(1): 93-110.

Kravchuk, R. S. and R. W. Schack (1996). "Designing effective performance-measurement systems under the Government Performance and Results Act of 1993." Public Administration Review **56**(4): 348-358.

Landry, R., N. Amara and M. Lamari (2001). "Climbing the ladder of research utilization evidence from social science research." Science Communication **22**(4): 396-422.

Latham, G. P., L. Borgogni and L. Petitta (2008). "Goal Setting andPerformance Management in the Public Sector." International Public Management Journal **11**(4): 385-403.

Lavis, J. N., A. D. Oxman, S. Lewin and A. Fretheim (2009). "SUPPORT Tools for evidence-informed health Policymaking (STP)." Health Res Policy Syst **7 Suppl 1**: I1.

Lavis, J. N., M. G. Wilson, A. D. Oxman, S. Lewin and A. Fretheim (2009). "SUPPORT Tools for evidence-informed health Policymaking (STP) 4: Using research evidence to clarify a problem." Health Res Policy Syst **7 Suppl 1**: S4.

Locke, E. A. and G. P. Latham (2002). "Building a Practically Useful Theory of Goal Setting and Task Motivation. A 35-Year Odyssey." American Psychologist **57**(9): 705–717.

MacFarlane, A., C. Barton-Sweeney, F. Woodard and T. Greenhalgh (2013). "Achieving and sustaining profound institutional change in healthcare: Case study using neo-institutional theory." Social Science & Medicine **80**: 10-18.

Macfarlane, F., M. Exworthy, M. Wilmott and T. Greenhalgh (2011). "Plus ça change, plus c’est la même chose: senior NHS managers’ narratives of restructuring." Sociology of Health & Illness **33**(6): 914-929.

Mark, M. M. and G. T. Henry (2004). "The Mechanisms and Outcomes of Evaluation Influence." Evaluation **10**(1): 35-57.

Meier, K. J., L. J. O'Toole, G. A. Boyne and R. M. Walker (2007). "Strategic management and the performance of public organizations: Testing venerable ideas against recent theories." Journal of Public Administration Research and Theory **17**(3): 357-377.

Monroe, K. R. and K. H. Maher (1995). "Psychology and rational actor theory." Political Psychology **16**(1): 1-21.

O'Toole Jr, L. J. (2000). "Research on policy implementation: Assessment and prospects." Journal of Public Administration Research and Theory: J-PART **10**(2): 263-288.

Ottoson, J. M. (2009). "Knowledge-for-action theories in evaluation: knowledge utilization, diffusion, implementation, transfer and translation." New Directions for Evaluation(124): 7-20.

Perry, J. T., T. A. Engbers and S. Jun, Y. (2009). "Back to the Future? Performance-Related Pay, Empirical Research, and the Perils of Persistence." Public Administration Review **69**(1): 39-51.

Rogers, E. M. (2003). Diffusion of Innovations, 5th Edition.

Ryan, R. M. and E. L. Deci (2000). "Self-determination theory and the facilitation of intrinsic motivation, social development, and well-being." American Psychologist **55**(1).

Schwartz, R. P. M. T., Evaluation, and Evidence-Based Policy. Mind the Gap: Perspectives on Policy Evaluation and the Social Sciences, 1, 121. (2010). Public Management Theory, Evaluation, and Evidence-Based Policy. Mind the Gap: Perspectives on Policy Evaluation and the Social Sciences. R. C. Rist. New Brunswick, USA, Transaction Publishers. **16**.

Scott, W. R., Ruef, M., Mendel, P. J., & Caronna, C. A. (2000). Institutional change and healthcare organizations: From professional dominance to managed care. Chicago, The University of Chicago Press.

Shiffman, J. (2007). Generating political priority for public health causes in developing countries: Implications from a study on maternal mortality. Generating political priority for public health causes in developing countries: Implications from a study on maternal mortality, CGD.

Shiffman, J. (2009). "A social explanation for the rise and fall of global health issues." Bulletin of the World Health Organization **87**(8): 608-613.

Streib, G. D. and T. H. Poister (1999). "Assessing the validity, legitimacy, and functionality of performance measurement systems in municipal governments." The American Review of Public Administration **29**(2): 107-123.

Tetroe, J. (2007). Knowledge translation at the Canadian Institutes of Health Research: a primer. Focus Technical Briefs. Ottawa, Canada, Canadian Institutes of Health Research.

Wang, X. (2002). "Assessing administrative accountability results from a national survey." The American Review of Public Administration **32**(3): 350-370.

Weiss, C. H. (1998). "Have we learned anything new about the use of evaluation?" The American Journal of Evaluation **19**(1): 21-33.

Weyland, K. (2005). "Theories of policy diffusion." World Politics **57**: 269-295.

Weyland, K. (2005). "Theories of policy diffusion - lessons from Latin American Pension Reform." World politics **57**(2): 262-295.
