# Supplementary material for: Characterizing performance improvement in primary care systems in Mesoamerica: A realist evaluation protocol
Source: Gates Open Res. 2018 Jan 3;2:1. [Version 1] doi: 10.12688/gatesopenres.12782.1 (PMC5801599; doi:10.12688/gatesopenres.12782.1)
Supplement: Supplementary file 2 [file gatesopenres-2-13842-s0001.tgz › 96bcf253-11fe-433d-b15b-df0c70cace40.docx]

**Interview guide for the MOH staff in Honduras and El Salvador**

**Let’s start by exploring the beginning of SMI. Tell me about the pre-existing political, policy, and programmatic conditions that led your country to the decision of participating in the Salud Mesoamerica Initiative (the initiative)?**

*Probes:*

- What role did the government priorities in 2010-11 played?
- Did the Central-American integration structures play any role? What/how?
- What influence did international/global commitments play? Why? Can you describe it? (Probe for the MDGs)
- What role, if any, did the involvement by the Bill & Melinda Gates Foundation and the Carlos Slim Foundation played in the government’s decision? Why? Please describe.
- How relevant was the involvement by the IADB as the entity managing the funds?
- Were there other factors in the environment that made SMI attractive to the government?

**Let’s discuss now the results-based financing approach used in SMI.**

- What aspects of this approach are appropriate?
  - Why?
- How about negative aspects?
  - Why?
- What are the effects of this RBF approach for the central level of the MOH?
- (If applicable) For the regional levels?
- For the local levels?

**Now let’s discuss the data collected through the external verification of performance (EVOP). Let’s start with the baseline.**

- What did the baseline show?

Probes:

- - How were the results received by the central-level staff?
  - What actions were taken by the central-level?
  - What was your reaction when you learned about the baseline results?
  - What was the role by the Institute of Health Metrics and Evaluation?
  - And by the IADB?
- What aspects of the EVOP are attractive for the MOH? Anything worrying?

**Let’s discuss now the evaluation at the end of phase 1.**

- What did the evaluation show?
  - - Give me examples of any surprises for you, both positive and negative.
    - How were the results received by the central-level staff?
    - What was the role by the Institute of Health Metrics and Evaluation?
    - And by the IADB?
- What aspects of the EVOP approach are appropriate?
  - Why?
- How about negative aspects?
  - Why?
- What are the effects of the EVOP for the central level of the MOH?
  - (If applicable) For the regional levels?
  - For the local levels?
  - How are these results disseminated?
- Is the MOH staff prepared to use the resulting EVOP data?
- What does the central-level do with the results? Regional levels (if applicable)? Local levels?
- What is the value-added for the MOH from this EVOP?

Probes:

- - What does the EVOP data do for you in your current role?
  - What else is missing from the EVOP data? Anything else need to be included?
- Have you received help in reviewing, analyzing or interpreting the EVOP data? From whom? (Probe for examples)
- What else could SMI do in the future to support the MOH in better using the EVOP data?

**Let’s talk about the future, once SMI ends.**

Do you think the EVOP needs to continue once the Initiative ends? Why?

What factors would determine the continuation of the EVOP?

If an external verification weren’t possible, should the MOH develop internal verification mechanisms? What would be the obstacles or challenges for this option?

# In-depth interview guide - Family Health Teams (FHT)

1. In 2011, where were you working? What was your role? *(Probe roles and responsibilities in the FHT and SMI)*
2. What are your roles and responsibilities in your current job?
3. What are the most common health issues in your municipality? *(Probe about health maternal and neonatal issues)*
4. What do you like the most about SMI? How has it helped?
5. As you know, SMI conducts performance evaluations; Your FHT has been positively assessed. How does it feel to have done well in the evaluation?
6. Let’s further explore this “feeling good” in the evaluation. Why does it feel good when the FHT demonstrates good performance or comes out “well evaluated”?
7. What do you feel when the result from an evaluation is favorable?
   1. Why?
8. In your FHT and in this health center many individuals work. What happens when an individual does not do his/her job well?
   1. What does the group do?
   2. Who takes care about these matters?
   3. What have you all learned from these situations?
   4. (*For each source of motivation* *mentioned*) I want to better understand that idea you just mentioned. Why is it produced? How does it feel when you do “that”?
9. What effects do you think that are produced from receiving *community recognition*?
10. Let’s discuss compensation now *(salary, payment, bonuses)* of this FHT/health center’s staff. How do you feel about such compensation? Does it truly represent the work done? How important is to feel that the salary recognizes the work? Why? *Give me an example*.
11. Some say that if one gives a bonus or better compensation, such a thing could reduce the internal drive for work. What do you think about this?
12. Folk seem to give importance to the regional meetings where you all discuss the results from the FHT/Health Center. Have you attended such meetings? What do you think about them? What is the use of attending those meetings?
13. Sometimes comparisons between FHTs with different performance are done in these meetings. Have you been to meetings where these comparisons are made? What do you think about that? What is the use for you all from attending these kinds of meetings? *(Probe the feelings and reactions from participating in benchmarking exercises).*
14. Have you participated in any of the Collaboratives? What has been your experience? (*Probe for motivation*) .
    1. How does it feel to participate?
    2. What do you feel?
    3. What is the use of these collaboratives?

# IHME Interview guideline

We will present some statements. Please give us your opinions about each of them.

1. “The act of performance measurement can have an influence on performance itself” –
   1. What do you think about this statement?
   2. Why?
   3. Can you give an example from your experience?
2. “External versus internal performance measurement have different influences on performance.”
   1. What do you think about this statement?
   2. Why?
   3. Can you give an example from your experience?
3. In your experience, how do country stakeholders in SMI perceive the process of external verification of performance? What about the results? How are they perceived?
   1. Why do you think that is?
   2. Examples?
4. In your experience, what were the intended uses and applications that country actors would make of the performance verification data collected by IHME? By the IADB? By SMI’s donors?
   1. Can you think of any other uses or applications by country actors?
5. Once results are collected, how does IHME explain the results of verification to country actors?
6. When IHME presented the baseline results in countries, what were some of the reactions that you witnessed in the audience?
   1. How does this differ from other instances in which IHME presents, for instance the results of impact evaluations?
   2. (If there is a difference) Why do you think there is a difference? Can you give me an example?
7. In your experience, how do country actors in Mesoamerica use external measurement to improve performance?
   1. Can you give an example from your experience?
8. “External measurement can be as equally powerful as financial incentives to drive country performance?”
   1. What do you think about this statement?
   2. Why?
   3. Can you give an example from your experience?

**Interview guide for IADB staff in Washington, Honduras and El Salvador**

**Let’s start by exploring the beginning of SMI. Tell me about the pre-existing political, policy, and programmatic conditions that led countries (or a specific country) to the decision of participating in the Salud Mesoamerica Initiative (the initiative)?**

*Probes:*

- What role did government priorities in played?
- Did the Central-American integration structures play any role? What/how?
- What influence did international/global commitments play? Why? Can you describe it? (Probe for the MDGs)
- What role, if any, did the involvement by the Bill & Melinda Gates Foundation and the Carlos Slim Foundation played in the government’s decision? Why? Please describe.
- How relevant do you think was the involvement by the IADB?
- Any other factors in the environment that made SMI attractive to the governments?

**Let’s discuss the results-based financing approach used in SMI.**

- What aspects of this approach are appropriate?
  - Why?
- How about negative aspects?
  - Why?
- What are the effects of this RBF approach for the ministries of health?
  - (If applicable) For the regional levels?
  - For the local levels?

**Now let’s discuss the data collected through the external verification of performance (EVOP).**

- How are EVOP results received once they are presented to country actors?
- What actions are taken by the government?
- What aspects of the EVOP are attractive for the ministries? Anything worrying?
  - What is the role played by the IADB in the EVOP process?
- What aspects of the EVOP approach are appropriate?
  - Why?
- How about negative aspects?
  - Why?
- What are the effects of the EVOP for the central level of the MOH?
  - (If applicable) For the regional levels?
  - For the local levels?
  - How are these results disseminated?
- Is the MOH staff prepared to use the resulting EVOP data?
- What does the central-level do with the results? Regional levels (if applicable)? Local levels?
- What is the value-added for the MOH from this EVOP?
- What else could SMI do in the future to support the ministries for better using the EVOP data?

**Let’s talk about the future, once SMI ends.**

Do you think the EVOP needs to continue once the Initiative ends? Why?

What options are there for the continuation of the EVOP?

If an external verification weren’t possible, should the MOH develop internal verification mechanisms? What would be the obstacles or challenges for this option?
