# Supplementary material for: Characterizing performance improvement in primary care systems in Mesoamerica: A realist evaluation protocol
Source: Gates Open Res. 2018 Jan 3;2:1. [Version 1] doi: 10.12688/gatesopenres.12782.1 (PMC5801599; doi:10.12688/gatesopenres.12782.1)
Supplement: Peer review history

# Peer review: Referee response for version 1

**DOI:** 10.21956/gatesopenres.13842.r26179

**Published:** 2018-03-05

**Authors:** Lisa R. Hirschhorn

**Affiliations:** 1 Feinberg School of Medicine, Northwestern University, Chicago, IL, USA

The authors have developed an in depth and well written description of the rationale behind and approaches to the protocol for a study using a realist evaluation approach to do an intern evaluation of AMI, a large multicounty accountability-driven intervention. The approach will complement the plan program evaluation being completed by IHME which is largely looking at explicit program defined outcomes. The description off SMI particularly for readers not as familiar with the structure is very helpful.

 The authors are clearly fluent in Realist Evaluation and familiar with many of the underlying theories which they use. However there is a lack of clarity of what the main focus is of this manuscript is describing and the use of the term “study” is often confusing as referring to different scopes of work.

 For example:

 Abstract:

 The initial sentence which reads “ This **study** presents the protocol for a **study **that uses a realist evaluation approach to develop a preliminary program theory that hypothesizes the interactions between context, interventions and the mechanisms that trigger outcomes. The program theory was completed through a scoping review of relevant empirical, peer-reviewed and grey literature; a sense-making workshop with program stakeholders; and content analysis of key SMI documents.” And then goes onto to say “This **study**”.

 In the text, the reviewer was still confused which study was being described (the development, the testing, et he evaluation leading to results including a refined program theory) and clarity would be helpful, including that the protocol describes work already done (development of the preliminary program theory) as well as how it will be applied in the future.

 In framing the manuscript in the text, later they then state “ This study addresses two research questions:  “(1) What are the effects of using supply-side financial incentives on the performance of the primary care systems in Honduras and El Salvador? How are those effects produced? Under what contextual factors are these effects produced in each country? And, (2) What are the effects of continuous external verification of performance in the two countries under study? How are those effects produced? Under what contextual factors are these effects produced in each country?

 While I assume that this use of the term: study” refers to the realist evaluation rather than the development of the program theory. For example in the section “ Study design” it states “. In this step the preliminary program theory will be tested, further developed, and validated or rejected.”

 Given the critical importance of the qualitative data to be collected through interviews, a bit more detail in how the interviewees will be sampled (site, individual, area in the respective countries)

 In their challenges part, it would be helpful to understand a bit more the limitations imposed by the 2 countries chosen from SMI for this study, and what characteristics differ from other SMI countries not chosen for this evaluation

 Minor:

 On page 8 in describing the program theory, I am curious that inputs are not explicitly called out as needed (and related to context) and that equity and effectiveness are also not explicit in the theory.

 Given the design of SMI and the underlying approach of Realist Evaluation, I was curious if the researchers had considered including community interviewer and or patients as critical to the success (and acceptability) of the intervention.

 Are they also planning to assess fidelity to the planned implementation (and adaptations implemented locally or at a national level) which could change the outcomes and be related to or change the mechanisms (as well as inform potential future adaptations.

I have read this submission. I believe that I have an appropriate level of expertise to confirm that it is of an acceptable scientific standard, however I have significant reservations, as outlined above.

# Peer review: Referee response for version 1

**DOI:** 10.21956/gatesopenres.13842.r26182

**Published:** 2018-01-30

**Authors:** Jean-Paul Dossou

**Affiliations:** 1 Centre de Recherche en Reproduction Humaine et en Démographie, CNHU/HKM, Cotonou, Benin; 2 Institute of Tropical Medicine of Antwerp, Antwerp, Belgium

This is a brilliant manuscript, among the best I have ever reviewed. The subject  is relevant and this paper will serve several academic and scientific purposes. 

 The following comments and questions may help in improving some minor points.

1. In which regards are El Salvador and Honduras contrasting cases? Can authors provide a brief comparison table showing in which dimensions those countries are considered contrasting cases?
2. Figure 2: Improve the display of the four squares of the "scalling-up of interventions" box.
3. Study design/1st paragraph
  Authors reported the following "we define each country’s primary care system as the unit of analysis". Can authors provide an operation definition/conceptual framework of "country's primary care system" within this protocol?
4. Data analysis/4th paragraph
  "preliminary program theory and the causal patterns identified." not "preliminary program thyeory and the causal patterns identified."
5. Data analysis/1st paragraph
  We suggest to authors to include "actors " in the "context, intervention, mechanism, and outcome " structure to have "intervention, context, actor, mechanism, and outcome (ICAMO) " like here (https://bmcpublichealth.biomedcentral.com/articles/10.1186/s12889-017-4322-8#CR42). Authors may also broaden the CMO configuration to consider the ICAMO configuration that may improve quality in the analysis and make a better and more explicit use of the role of actors in the analysis.

I have read this submission. I believe that I have an appropriate level of expertise to confirm that it is of an acceptable scientific standard.

# Response

**Published:** 2018-01-30

**Authors:** Wolfgang Munar

**Affiliations:** George Washington University, USA

Dr. Dossou: Les auteurs apprécient vos commentaires. Merci beaucoup.

We will consider them all, while editing the paper.

# Peer review: Referee response for version 1

**DOI:** 10.21956/gatesopenres.13842.r26180

**Published:** 2018-01-12

**Authors:** Daniel H. Kress

**Affiliations:** 1 RTI International , Seattle, WA, USA

I recommend publication with minor revisions. 

 As this is a study protocol as opposed to the actual study, there are no datasets at this time so the answer to the question, "Are the datasets clearly presented in a useable and accessible format" can only be partly or actually NA since the data will be collected using the study protocol that is proposed for publication and eventually that will be carried out to assess the impact of SMI. 

 Overall, I find this to be a thorough and carefully thought out study protocol that will provide important insights into how the results produced by SMI were actually created.  As such, this study protocol will shed important insights into how a large, complex intervention across multiple countries and over time produced the quite astounding results that marked the success of SMI. Even as we have seen the positive results from the regular evaluations and can easily see the quite significant improvements countries that are part of this initiative have registered, important questions as to what factors actually drove the impact seen remain only partially answered. This study will shed important light on these questions.

 I only have a few minor quibbles regarding the article.

1. The authors use PBF and RBF almost interchangeably and sometimes use both terms. I think it might be less confusing to the reader to define terms up front and then use one term.
2. Page 3, paragraph 8, says that studies on the effects of RBF on large scale system reforms are largely absent. Later on the authors cite a systematic review. In fact, there have been a number of systematic reviews of RBF programs beyond the one cited. For example, Andy Oxman has several papers that review (critically) the experience with RBF. Miller and Singer (2013) is another.
3. I also think that in the area of RBF, it's important to not focus only on LMIC experience as RBF is an instrument that has been used and is being used extensively. The Quality and Outcomes Framework (QOF) in the UK NHS is an example. Peter Smith has a number of papers that reviews that experience and Cheryl Cashin and Peter Smith have a paper on how RBF links to the larger issue of Strategic Purchasing.
4. Perhaps my strongest comment is on page 7, paragraph 6, regarding the program theory section. I think it's quite possible to formulate a hypothesis that SMI was not primarily a classic extrinsic financial incentive program but possibly much more an extrinsic non pecuniary program where the rewards were doing well amongst your peers. When you look at the incentive rewards, its difficult to see how such relatively small financial rewards could incent behavior. The counterpoint to this argument might be that the funding provided by the SMI donors was flexible and in these heath systems flexible funding is often rare and highly prized but that too is an issue deserving of further investigation. However, if the funding is small and relatively insignificant, the question is then what drove the behavior and actions taken. A factor worthy of investigation is the SMI approach of engaging multiple countries in a form of joint competition. Ministers of Health were all engaged on SMI and there is some anecdotal evidence that the approach of having them compete together, each trying to attain the targets they set for their own country, created a form of competition or at least a common forum where not performing well would be seen as a distinct negative outcome, thereby conferring strong incentives for them to perform well or endeavor to make sure their health system performs strongly. This kinds of peer effects are known to be powerful in behavioral economics and so we should look for them in this study as well.  

I have read this submission. I believe that I have an appropriate level of expertise to confirm that it is of an acceptable scientific standard.

# Response

**Published:** 2018-01-30

**Authors:** Wolfgang Munar

**Affiliations:** George Washington University, USA

Dear Dan,

The entire team read your comments. We appreciate them enormously and will tackle them in our upcoming edited and final version.

Thanks a lot,

Wolfgang on behalf of the team.
